# Evidence That β1-Integrin Is Required for the Anti-Viability and Anti-Proliferative Effect of Resveratrol in CRC Cells

**DOI:** 10.3390/ijms23094714

**Published:** 2022-04-25

**Authors:** Aranka Brockmueller, Parviz Shayan, Mehdi Shakibaei

**Affiliations:** 1 Musculoskeletal Research Group and Tumor Biology, Chair of Vegetative Anatomy, Faculty of Medicine, Institute of Anatomy, Ludwig-Maximilian-University Munich, Pettenkoferstr. 11, D-80336 Munich, Germany; aranka.brockmueller@med.uni-muenchen.de; 2 Department of Parasitology, Faculty of Veterinary Medicine, University of Tehran, Tehran 141556453, Iran; pshayan@ut.ac.ir

**Keywords:** tumor microenvironment, β1-integrin, resveratrol, RGD peptides, proliferation, NF-kB, bacitracin

## Abstract

The β1-integrin receptor is broadly expressed on tumor and other cells in the tumor microenvironment (TME), and is an unfavorable prognostic factor for cancers. Nature-derived resveratrol has preventive and apoptotic effects on tumors, but whether resveratrol can exert its suppressive actions on TME-induced tumorigenesis through β1-integrin on the surface of CRC cells is still unknown. HCT116 or SW480 cells were exposed to inhibitory antibodies against β1-integrin, bacitracin (selective β1-integrin inhibitor), integrin-binding RGD (Arg-Gly-Asp) peptide, and/or resveratrol. We evaluated the anti-tumor actions and signaling impacts of resveratrol in colorectal cancer (CRC)-TME. We found that resveratrol completely altered the β1-integrin distribution pattern and expression on the surface of CRC cells in TME. Moreover, resveratrol down-regulated CRC cell proliferation, colony formation, viability, and up-regulated apoptosis in a concentration-dependent way. These actions of resveratrol were antagonized mainly by inhibitory antibodies against β1-integrin but not β5-integrin, and by an integrin-binding RGD peptide but not by RGE peptide, and by bacitracin in TME. Similarly, resveratrol-blocked TME-induced p65-NF-kB and its promoted gene markers linked to proliferation (cyclin D1), invasion (focal adhesion kinase, FAK), or apoptosis (caspase-3), were largely abrogated by anti-β1-integrin or RGD peptide, suggesting that β1-integrin is a potential transmission pathway for resveratrol/integrin down-stream signaling in CRC cells. The current results highlight, for the first time, the important gateway role of β1-integrins as signal carriers for resveratrol on the surfaces of HCT116 and SW480 cells, and their functional cooperation for the modulatory effects of resveratrol on TME-promoted tumorigenesis.

## 1. Introduction

Colorectal cancer (CRC), mainly due to epigenetic alterations, has become widely recognized as being among the most common aggressive adenocarcinomas causing cancer deaths worldwide, and unfortunately, the incidence of CRC is increasing [1,2,3]. In current statistics, CRC ranks third in the number of new cancer incidents as well as in the total of patients who have died from cancer [4]. This high value shows the importance of developing complementary and adequate treatment strategies. The different stages of cancer development have been known for years and are of great relevance because each stage offers the opportunity to intervene in the tumor process. Tumors also have the ability to recruit seemingly normal cells to help them establish a specific tumor microenvironment (TME) [5], underscoring cancer as a complex, multi-stage disease that requires multi-targeting therapy.

In recent years, several papers have highlighted the relevance of the TME in CRC progression, proliferation, and metastasis. However, the extracellular matrix (ECM) compounds take a central role in this context. Among the components of the ECM responsible for adhesion, collagens, and proteoglycans are some of the most important matters controlling cancer-related processes at each stage of tumorigenesis [6,7,8]. Therefore, the TME plays a central role in tumor research and also in the generation of novel drugs for the therapeutic intervention of tumors. A multicellular TME consists of a variety of active cells (immune cells, tumor-associated fibroblasts, endothelial cells, pericytes, adipocytes, and other stromal cells) as well as many ECM proteins and enzymes produced by tumor cells and stromal cells [5,9,10]. This complex network of ECM in the TME plays a fundamental role in tumor progression and proliferation, and is partially discussed as a specific biomarker for tumor malignancy [11,12,13]. 

An intense interaction exists between cells and ECM in TME that is critical for tumor cell progression. One of the most important cell surface receptor families that plays a major role in TME in cell-matrix interaction and simultaneously functions as signaling molecules is the integrin family [8,9], thus representing a very important and attractive therapeutic target in TME. It is known that integrins are transmembrane glycoproteins, composed of non-covalent, heterodimeric complexes of an α- and a β-chain, that can signal bidirectionally upon binding to their ECM compounds, and thus regulate a number of important biological activities [14,15,16,17]. Association of integrins with the ECM component causes an active interaction between integrins and cytoskeleton, and signaling complexes that induce the assembly of cytoskeletal filaments. Modulation of cytoskeletal filaments further activates integrins to bind more matrix, resulting in an enhanced feedback system. Furthermore, this interaction induces specific adaptor molecules to form focal adhesion aggregates. Thus, integrins as functional integrators, enable binding and also signal transduction between ECM and cytoskeletal proteins, and these very highly orchestrated complexes are designated as focal adhesion sites [18], highlighting their role in angiogenesis, growth, migration, and invasion of tumors. Consequently, preclinical and clinical works displayed interventions in tumor progression by integrin antagonists [19,20]. Notably, distinct integrins on tumor cells have been found to play essential functional roles in the progression of diverse tumors, such as αvβ3, αvβ5, α5β1, α6β4, and α4β1, which serve as a prognostic marker for early-stage overall survival in TME, suggesting that integrin receptors act as regulators of tumor survival and progression [21,22,23,24,25,26]. 

Natural plant compounds such as resveratrol can promote health, and support both disease prophylaxis and co-treatment of many diseases. This is possible because they are multi-targeting agents having the ability to modulate various signaling pathways. Resveratrol (3,5,4′trihydroxy-trans-stilbene) has been identified as a plant polyphenol in berries, grapes, red wine, soy, and peanuts [27]. However, with respect to cancer cells, resveratrol showed anti-tumor, anti-mutagenic, chemopreventive, anti-oxidative, and anti-inflammatory activities. In fact, resveratrol is known to exert these properties in CRC through modulation of various signaling pathways such as CamKKB/AMPK, talin-FAK, Sirt1, NF-kB, and also glycolysis, and pentose phosphate pathway [28]. Moreover, the cysteine-rich domain of integrin is assumed to hold the binding site for resveratrol. Indeed, resveratrol has previously been reported to bind to the integrin αvβ3 receptor in breast cancer cells, thereby activating ERK1/2 and AMPK to induce COX-2 accumulation, and subsequently, p53-dependent apoptosis [29]. In SW480 CRC cells, the αvβ3-integrin receptors have been shown to be even involved in both resveratrol-uptake by the CRC cells as well as resveratrol-induced apoptosis [26].

Although the anti-tumor activity of some active components via αvβ3-, αvβ5-, and β1-integrins in many different tumors has been reported more frequently in the past, the anti-tumor activity of resveratrol in association with β1-integrin receptors in CRC cells is still unknown. In our previous study, we found that resveratrol attenuated TME-induced CRC cell growth and migration [30]. Now, we addressed whether resveratrol can interact with β1-integrin on the surfaces of HCT116 or SW480 cells and exert its anti-proliferative, anti-viable, and pro-apoptotic effects in an in vitro TME model.

## 2. Results

In our previous research, we have demonstrated that resveratrol attenuates pro-inflammatory TME-induced CRC cell growth and migration [30], and what we want to further evaluate is regarding the specificity and details of relevant pathways of these interactions. In this work, we assessed the potential of resveratrol to act on β1-integrin receptors at the surface of CRC cells to drive its anti-tumor-related cellular actions in an in vitro 3D-TME model. 

### 2.1. Resveratrol Alters the Expression and Distribution Pattern of β1-Integrin Receptors on the Surface of HCT116 and SW480 Cells in the TME

To analyze potential functional targets of the anti-tumorigenesis effect of resveratrol in the TME microarchitecture in CRC cells, we initially left the HCT116 and SW480 cells in the TME untreated or treated with resveratrol (5 µM), and with or without anti-β1-integrin antibody (2 µg/mL). Cells were immunolabeled with β1-integrin antibody, and DAPI counterstaining was carried out to reveal the cell nuclei.

**Figure 1:**
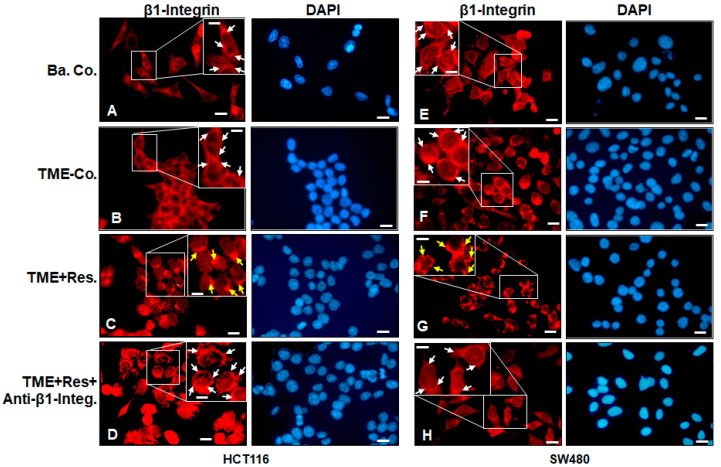
Immunofluorescence microscopy visualization of β1-integrin receptors under resveratrol challenge on the surface of HCT116 and SW480 cells in the TME. β1-integrin immunolabeled (white arrows) and DAPI-stained nuclei from untreated basal control (**A**,**E**); TME-grown (**B**,**F**); resveratrol-treated (5 µM) (**C**,**G**), and further addition of anti-β1-integrin (2 µg/mL) (**D**,**H**) HCT116 and SW480 cells. Yellow arrows = change in distribution pattern of β1-integrin receptors. Microscope: Leica DM 2000. Magnification ×600; scale bar = 30 µm. Insets: magnification: ×1200; scale bar = 15 µm.

Immunolabeling study by immunofluorescence microscopy in TME control cultures showed that HCT116 and SW480 cells had strong uniform and homogeneous cell membrane labeling for β1-integrin (Figure 1B,F), compared with HCT116 and SW480 cells in the basal control (Figure 1A,E) without TME. Interestingly and surprisingly, we found that treatment of TME cultures with resveratrol caused a remarkable change in the expression and distribution pattern of β1-integrin receptors in HCT116 and SW480 cells (Figure 1C,G, yellow arrows). Indeed, the expression of integrin receptors became punctate rather than homogeneous on the surface of the cells. In contrast, simultaneous treatment of HCT116 and SW480 cells in TME with resveratrol and a specific anti-β1-integrin blocked the resveratrol-induced punctate distribution pattern of β1-integrin receptors again, i.e., the expression and distribution pattern of β1-integrin receptors on the surface of tumor cells remained homogeneous (Figure 1D,H), as in the TME control (Figure 1B,F). These findings are in accordance with other reports demonstrating that integrins are overexpressed in the cell membranes of mitotic-active and various tumor cells [31]. Taken together, these results underline the critical role of β1-integrin receptors as one of the first transmission molecules of resveratrol signaling pathway in the TME, and this modulatory effect of resveratrol on the distribution of β1-integrin receptors on the CRC cell membrane was not cell line-specific.

### 2.2. Resveratrol Blocks TME-Stimulated Expression of β1-Integrin, but Not β5-Integrin in CRC Cells

**Figure 2:**
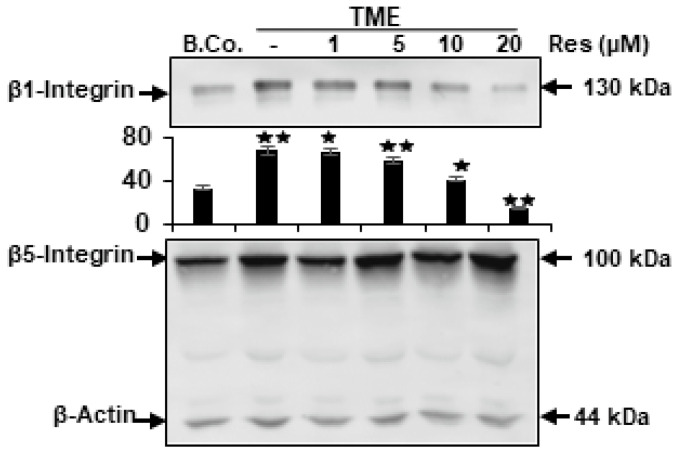
The impact of resveratrol on TME-triggered expression of β1- and β5-integrin. HCT116 cells in alginate matrix in basal control (B. Co.) or in TME were untreated or TME treated with diverse dosages of resveratrol (1, 5, 10, 20 μM) for 10–14 days as outlined in Materials and Methods. Immunolabeling of cell lysates was conducted by Western blotting with antibodies against β1- and β5-integrin. Data are originated from three separate assays, and β-actin was used as a reference. *Y*-axis: densitometric units confirming Western blot results. * *p* < 0.05, ** *p* < 0.01 compared with TME control.

It has been previously reported that integrin receptors in the TME are overexpressed and play an essential role in tumor cell progression, survival, and as markers for tumor prognosis [23]. We wanted to investigate the action of resveratrol on up-regulated and activated integrin expression in the TME. For this purpose, HCT116 cells were cultured in 3D-alginate by themselves (basal control) or co-cultured as 3D-alginate culture in the TME treated with various doses of resveratrol (0, 1, 5, 10, and 20 µM) for 10–14 days, as outlined in the Materials and Methods. Western blotting results showed that the expression of β1-integrin was markedly elevated in TME-control cells compared to basal control. However, treatment of HCT116 cells in the TME with resveratrol clearly showed a dose-dependent down-regulation of β1-integrin but not β5-integrin compared with TME-control (Figure 2). Resveratrol exerts a profound dose-dependent action on TME-activated β1-integrin expression in HCT116 cells. To note, at a concentration of 10 μM resveratrol, β1-integrin expression was markedly reduced, and at a concentration of 20 μM of resveratrol, β1-integrin was largely blocked compared with TME-control (Figure 2). Importantly, the concentration of resveratrol (5 µM) used as a working dose and the timing of treatment had marginal effects on β1-integrin expression. This was confirmed by quantitative densitometry. Indeed, there was no apparent effect of added resveratrol on the expression of β5-integrin, underscoring the role of β1-integrin in resveratrol’s specific signal transmission. Taken together, these data indicate a critical role of β1-integrin for TME-inducing tumorigenic effects in CRC cells, and further highlight that resveratrol suppresses, at least in part, TME-stimulated progression of HCT116 cells primarily via gateway β1-integrin receptor and down-stream β1-integrin signaling pathway.

### 2.3. β1-Integrin Serves as a Signal Transmission Receptor of Resveratrol in HCT116 and SW480 Cells

HCT116 or SW480 cultures were treated with resveratrol (0, 1, 5 µM) in the presence or absence of IgG (1, 2, 5 µg/mL), anti-β1-integrin (1, 2, 5 µg/mL), anti-β5-integrin (5 µg/mL), RGD peptide (1, 2, 5 µM), RGE peptide (5 µM), or bacitracin (1, 5 µM) for 10–14 days.

#### 2.3.1. Repression of β1-Integrin by Antibody Inhibits the Blocking Effect of Resveratrol on TME-Promoted Viability of CRC Cells

To investigate whether β1-integrin signaling is involved in resveratrol-induced anti-proliferative activity in HCT116 or SW480 cells in TME, the CRC cells were grown in 3D-alginate as basal control (Co.) or in TME control, or TME was treated with resveratrol (1, 5 µM), or anti-mouse IgG (served as the negative control) (1, 2, 5 µg/mL), or inhibitory anti-β1-integrin antibody (1, 2, 5 µg/mL) by itself, or were co-treated with 5 µM resveratrol and anti-mouse IgG (1, 2, 5 µg/mL), or anti-β1-integrin (1, 2, 5 µg/mL). Cell proliferation was examined by MTT assay as detailed in Materials and Methods. 

**Figure 3:**
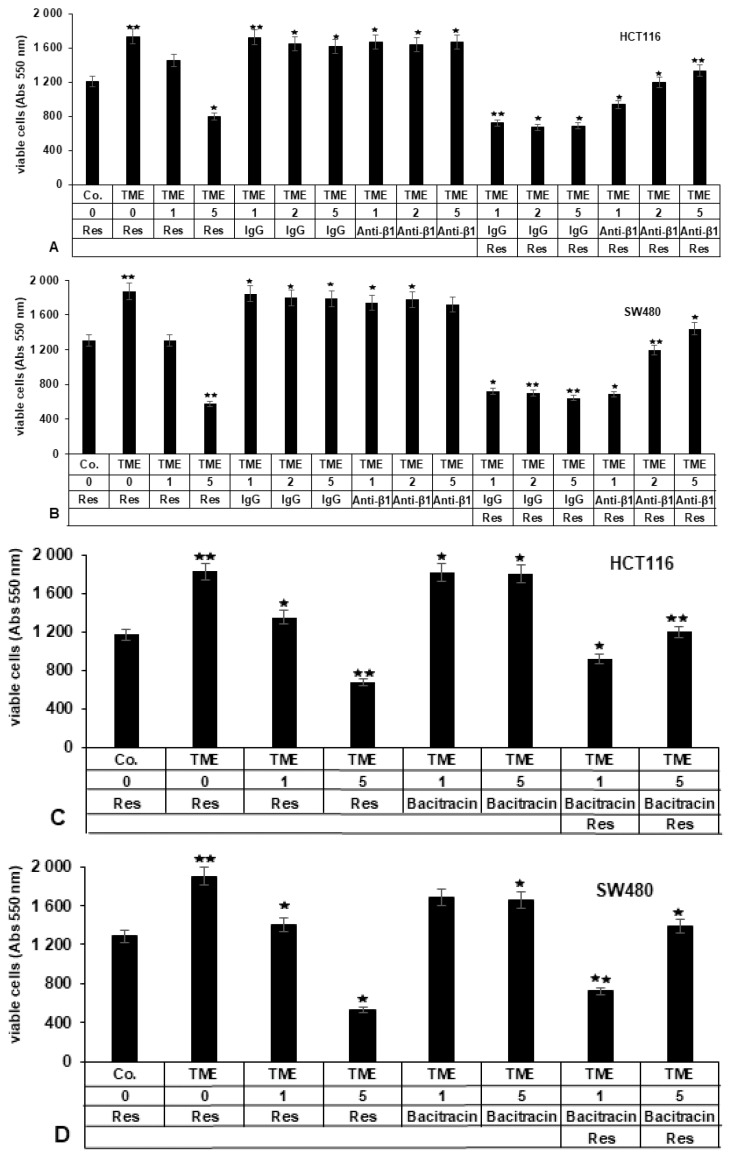

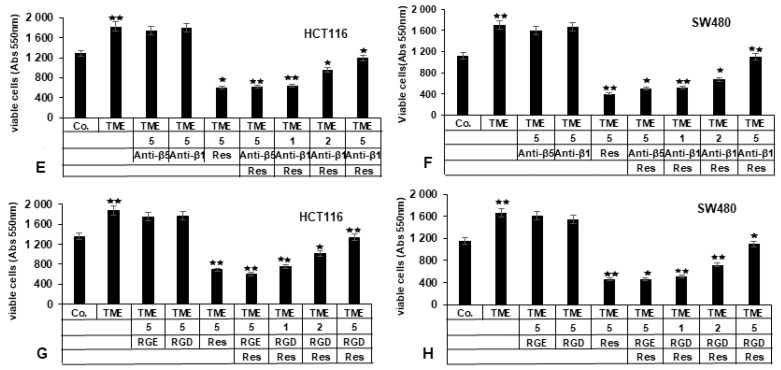
The importance of β1-integrin for resveratrol-mediated down-modulation of tumor cell viability: (**A**,**B**) CRC cells (HCT116, SW480) from basal control (Co.) or TME control were not treated or treated with resveratrol (1, 5 µM) in the presence or absence of IgG (1, 2, 5 µg/mL), the blocking anti-β1-integrin antibody (1, 2, 5 µg/mL); (**C**,**D**) bacitracin (1, 5 µM); (**E**,**F**) the blocking anti-β1-integrin antibody (1, 2, 5 µg/mL), anti-β5-integrin (5 µg/mL); (**G**,**H**) RGD peptide (1, 2, 5 µM), RGE peptide (5 µM) for 10–14 days. Tumor cell survival and, indirectly, proliferation were assessed by MTT assay. Relative to TME control, * *p* < 0.05 and ** *p* < 0.01.

TME significantly increased the viability and thus indirectly, the proliferation of HCT116 and SW480 cells, as evidenced by the fact that the measurement rate of viable cells for both cell lines was more than one-third higher in TME than in the basal control (Figure 3A,B). However, resveratrol significantly and concentration-dependently suppressed TME-enhanced viability in both CRC cell lines by around 19% and 55% in HCT116 as well as 32% and 69% in SW480 cells at 1 or 5 µM resveratrol compared with TME control. Treatment with IgG or anti-β1-integrin (Anti-β1) by itself did not significantly suppress cell proliferation in HCT116 or SW480 cells compared to untreated TME control cultures (Figure 3A,B). Of note, in opposite to control co-treatment with IgG and resveratrol, which reduced CRC cell viability by around 58% in HCT116 and 64% in SW480, co-treatment of CRC cells with inhibitory anti-β1-integrin antibody and resveratrol significantly abrogated resveratrol-induced inhibition of proliferation in tumor cells in a concentration-dependent way, so that at a concentration of 5 µg/mL anti-β1 and 5 µM resveratrol, only around 20% (HCT116) and 25% (SW480) respectively, less viable cells were measured than in the TME control (Figure 3A,B). These results are consistent with other findings stating that other integrins such as integrin αvβ3 are able to bind resveratrol on the surface of breast tumor cells [32]. Collectively, these data underline that the β1-integrin receptor is actively involved in the anti-viability and anti-proliferative effect of resveratrol on the surface of CRC cells (HCT116 and SW480). Furthermore, the involvement of β1-integrin in the suppressive effect of resveratrol on CRC cell viability and proliferation was not cell line-specific.

#### 2.3.2. Bacitracin (β1-Integrin Inhibitor) Suppresses the Inhibitory Impact of Resveratrol on TME-Induced Viability in CRC Cells

To confirm that β1-integrins are actively involved as surface receptors for the resveratrol signaling pathway on TME-promoted viability and thus indirect proliferation of CRC cells, HCT116 or SW480 cells were treated with resveratrol (0, 1, 5 µM) in the presence or absence of bacitracin (1, 5 µM), a selective pharmacological inhibitor of β1-integrin and β7-integrin-mediated cell adhesion [33], and evaluated by MTT assay. As demonstrated in Figure 3C,D, TME indirectly stimulated the proliferation of CRC cells in alginate beads, with over a third more viable CRC cells (HCT116, SW480) in the TME control than in the basal control. However, resveratrol inhibited TME-induced viability of cells in a concentration-dependent fashion by decreasing cell viability around 23% and 67% in HCT116 as well as 27% and 59% in SW480 at 1 or 5 µM resveratrol, compared to TME. However, there was no significant difference between the bacitracin treatment by itself and TME control group. Notably, bacitracin suppressed resveratrol-induced inhibition of HCT116or SW480 cell viability in a concentration-dependent way (Figure 3C,D), visible by a reduction of viable cells by 32% (HCT116) and 27% (SW480) respectively, compared to TME, at a co-treatment with 5 µM bacitracin and 5 µM resveratrol. These data confirm the results from Figure 3A,B and underline that resveratrol-promoted inhibition of CRC cell viability is, at least in part, dependent on β1-integrin signaling pathway, and this was not cell line specific.

#### 2.3.3. RGD-Peptide, Similar to Anti-β1-Integrin, in Opposite to RGE Peptide or Anti-β5-Integrin, Inhibits Resveratrol-Suppressed TME-Induced Viability in CRC Cells

To further support the specific role of β1-integrin in signal transmission for resveratrol in HCT116 or SW480 cells, they were alternatively treated with resveratrol (5 µM) by itself or co-treated with anti-β5-integrin (5 µg/mL), or anti β1-integrin (1, 2, 5 µg/mL) (Figure 3E,F) or with an integrin inhibitor RGD peptide (1, 2, 5 µM), or control RGE-peptide (5 µM) (Figure 3G,H). Cell viability and thus indirectly, proliferation was investigated by MTT test as outlined in Materials and Methods. TME-induced viability of HCT116 as well as SW480 cells (over one third more viable CRC cells in TME control than in basal control) was clearly suppressed at a concentration of 5 µM resveratrol by around 67% (3E) and 64% (3G) respectively in HCT116, and 77% (3F) and 76% (3H) respectively in SW480, compared to TME as demonstrated before. To note, the RGD peptide reduced cell viability by around 27% in HCT116 and 32% in SW480 when co-treated with 5 µM RGD and 5 µM resveratrol, similar to the blocking antibody to β1-integrin (by around 26% in HCT116 and 33% in SW480 when co-treated with 5 µg/mL anti-β1-integrin and 5 µM resveratrol), in opposite to RGE peptide or anti-β5-integrin, and thus significantly blocked resveratrol-inhibited viability of CRC cells in a concentration-dependent way (Figure 3E–H), compared to TME control. Taken together, these data clearly demonstrate that β1-integrin and the β1-integrin signaling pathway, at least in part, is a potential receptor transfer pathway for the anti-tumor effect of resveratrol on the tumor cell membrane, and this was not cell line-specific, due to reproducibility in different CRC cell lines (HCT116, SW480).

### 2.4. β1-Integrin Signaling Pathway Is Involved in Resveratrol-Modulated TME-Induced Colony Formation in HCT116 Cells

**Figure 4:**
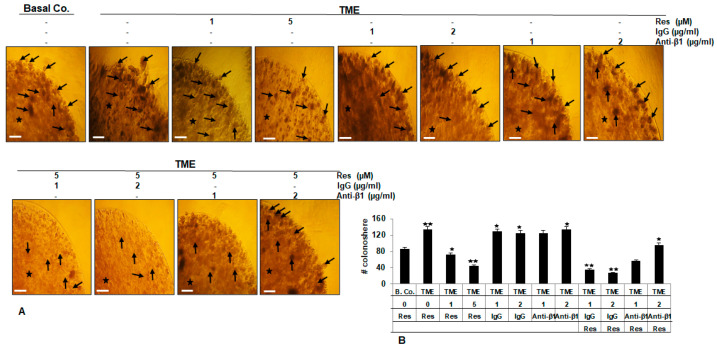
The implication of β1-integrin in resveratrol-mediated down-modulation of tumor cell colony assembly in TME: (**A**) HCT116 cells from basal control (Basal Co.) or untreated TME or TME were treated with different concentrations of resveratrol (1, 5 µM) in the presence or absence of IgG (1, 2 µg/mL) or blocking anti-β1-integrin antibody (1, 2 µg/mL) for 10–14 days as indicated in Materials and Methods; (**B**) Colonies (black arrows) were quantified by scoring 20 different microscopic patches. Microscope: Zeiss Axiovert 40 CFL. Magnification (A): ×24, bar = 0.2 mm. “star” = Alginate. * *p* < 0.05, ** *p* < 0.01 compared with TME control.

To evaluate further effects of β1-integrin receptor on resveratrol’s anti-tumor mechanisms, colony formation [34] of HCT116 cells, an essential and prominent feature of tumor cells, was performed in 3D-alginate TME cultures with resveratrol in the absence or presence of IgG or anti-β1-integrin, as outlined in Materials and Methods. Resveratrol by itself suppressed the colonosphere development of HCT116 (Figure 4A,B) in a dose-dependent mode. To note, TME initiated the quantity of colonosphere formations in CRC cells compared to that in basal control cultures of HCT116 cells (Figure 4A,B), indicating the pro-tumor role of TME in initiating the aggressiveness of CRC cells. In contrast, it was seen that there was no effect of IgG and anti-β1-integrin alone on colonosphere formation in CRC cells. Interestingly, only anti-β1-integrin, but not IgG, abolished the anti-colonosphere formation effects of resveratrol, so that the number of colonospheres formed in the 3D-alginate TME culture was similar to that in the control TME (Figure 4A,B). Thus, resveratrol has promising anti-CRC efficacy by inhibiting cell colony formation via β1-integrin.

### 2.5. β1-Integrin Is Required for Anti-Tumor Effects of Resveratrol in CRC Cells 

**Figure 5:**
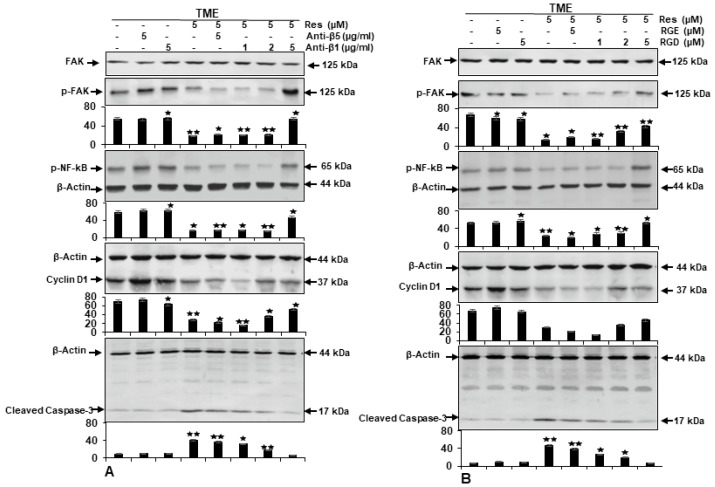
β1-integrin is required for anti-tumor effects of resveratrol. TME-HCT116 cells were treated with resveratrol (5 µM) in the presence or absence of anti-β1- (1, 2, 5 µg/mL), or anti-β5- integrin (5 µg/mL) (**A**) or of RGD (1, 2, 5 µM), or RGE (5 µM) peptide (**B**) as mentioned in Materials and Methods. Western blot samples were probed with antibodies against FAK, p-FAK, p-NF-kB, cyclin D1, and activated caspase-3. In addition, anti-β-actin was used as a loading control. *Y*-axis: densitometric units confirming Western blot results. * *p* < 0.05, ** *p* < 0.01 compared with TME control.

To more specify and confirm one of the primary functional proteins that recruits resveratrol to the surface membranes of HCT116 cells in TME, which has not been fully elucidated yet, the following studies were performed. HCT116 cells in 3D-alginate TME were provoked with or without an appropriate neutralizing antibody against β1-integrin (1, 2, 5 μg/mL), or antibody against β5-integrin (5 µg/mL) (Figure 5A), with or without an RGD peptide (1, 2, 5 µM), or RGE peptide (5 µM) (Figure 5B) for 10–14 days, as detailed in the Materials and Methods. Samples were tested by Western blotting with antibodies against FAK, p-FAK, (downstream of β1-integrin), pro-inflammatory transcription factor p65-NF-kB, cyclin D1 (proliferation), and activated caspase-3 (apoptosis). Immunoblotting of TME control cultures, similar to anti-β5- or β1-integrin, or to RGD-, or RGE-peptides by themselves, showed high expression of p65-NF-kB and p-FAK, cyclin D1 from HCT116 cells, and very low expression of activated caspase-3 (Figure 5A,B). As demonstrated in Figure 5A,B, treatment of HCT116 cells in TME with resveratrol resulted in down-regulation of p-FAK, p65-NF-kB, and cyclin D1 but a significant up-regulation of activated caspase-3 (Figure 5A,B). In contrast, we found that in combination with resveratrol and neutralizing antibodies against β1-integrin or resveratrol and RGD peptides, the effect of resveratrol on the above-stated proteins was revised in a concentration-dependent way (Figure 5A,B). However, the β5-integrin antibody or an inactive RGE peptide had no inhibitory effect on this process. Collectively, because of this, the down-regulatory actions of resveratrol on HCT116 cell proliferation are linked with the regulation of β1-integrin dependent FAK-, NF-kB-, and cyclin D1-signaling.

## 3. Discussion

Recently, accumulated evidence showed that tumor proliferation, malignancy, and growth may be motivated by ECM components and a variety of active paracrine interactions between stromal cells and tumor cells within the pro-inflammatory TME [30,35]. Consequently, a combination of anti-tumor and anti-stromal therapies has become an important research focus to develop new therapies for tumor treatment [35]. Therefore, in this research, we tested the possible contribution of the specific ECM adhesion receptor β1-integrin on the surface of HCT116 and SW480 cells as an initial transmitter for resveratrol-mediated anti-tumorigenesis signaling in TME.

It is widely accepted and, like others we have previously shown, that resveratrol, as a naturally occurring multi-targeting agent with biological activities, is extensively investigated with beneficial properties (anti-oxidative, anti-inflammatory, anti-microbial) in several biomolecular systems. Moreover, it is associated with a range of anti-cancer activities by directing multiple signaling pathways linked with cancer initiation, promotion, angiogenesis, and metastasis, as well as induction of apoptosis in various tumors [28,30,36,37,38,39]. However, the initial cellular-level interactions of resveratrol in CRC cells are incompletely understood, and a cellular receptor site of resveratrol signaling initiation has not been fully described yet.

Integrins are heterodimeric transmembrane proteins that have the ability to functionally connect cells to their specific microenvironment. Furthermore, they have been described as a family of cell membrane receptors involved in almost all functions and properties of many different tumors [14,18]. β1-integrin is capable of forming most of the junctions of multiple receptor complexes and thereby functionally associates with other subcellular signaling pathways, stimulating a number of genes and kinases such as FAK, which then leads to stimulation of cell adhesion, proliferation, and invasion [19,40]. Therefore, attempts have been made to develop targeting drugs directed against integrins. However, the results of treatment with these drugs in cancer therapy are very limited so far, as the specific integrin inhibitors are not approved for clinical use [19,41] yet.

We wondered whether our pro-inflammatory CRC-TME model induces β1-integrin expression in the HCT116 or SW480 cell line and, if so, whether this phenomenon is specifically modulated by resveratrol, which would be important for a therapeutic molecular experiment of tumor fate. At first, our immunofluorescence microscopy results showed that the expression of β1-integrin was significantly up-regulated in the HCT116- or SW480-TME compared to HCT116 or SW480 cells from the basal control environment. More interestingly, treatment of CRC cells with resveratrol changed the distribution pattern of β1-integrin receptors on the cell surface from homogeneous and uniform to a punctate. Indeed, it must be emphasized here that the alteration of the β1-integrin expression pattern by resveratrol observed is not an activation but rather a modulation of the β1-integrin receptors, which may facilitate the uptake or transduction capacity of resveratrol’s signals by the β1-integrin receptors. Moreover, it should be noted that co-treatment of HCT116 or SW480 cells with resveratrol and an anti-β1-integrin (neutralizing antibody) in the TME system abolished the resveratrol-induced change in the distribution pattern of β1-integrin receptors on the surface of both CRC cells. This shows that A) resveratrol modulates β1-integrin receptors for its signaling on the surface of tumor cells and B) resveratrol needs functional β1-integrin receptors, at least in part, to fully exert its anti-carcinogenic effect in CRC cells.

We further demonstrated that resveratrol significantly suppressed the viability and thus indirectly, proliferation of HCT116 and SW480 cells, and this suppression could be prevented by function-blocking antibodies to β1-integrin or treatment with RGD, or β1-integrin inhibition by specific inhibitor (bacitracin). Moreover, resveratrol was shown to inhibit TME-stimulated colony formation of HCT116 cells, and this inhibition was reversed by anti-β1-integrin. These results clearly suggest a critical, mediating, and important role of β1-integrin as an adhesion receptor and signaling molecule in TME-promoted proliferation and colony formation of CRC cells, and for the resveratrol signaling pathway in cellular changes, too. These results further suggest that pro-inflammatory TME-dependent up-regulation of β1-integrin is more likely a feature of CRC cells. Furthermore, this is consistent with other studies having shown that other types of integrin, such as β6-integrin, are sparsely expressed in normal intestinal epithelium but are highly up-regulated in the TME or by exogenous pro-inflammatory cytokines and act as tumor promoters [42,43]. More interestingly, these overall data are also concordant with earlier work by Lin et al. outlining that antibody against integrin αvβ3, but not αvβ5, or RGD peptide specifically impede the initiation of ERK1/2- and p53-dependent resveratrol-induced apoptosis in human breast cancer cells MCF-7 or MDA-MB231, thereby supporting the notion that integrin αvβ3 carries a receptor site for resveratrol [24]. In addition, αvβ3-integrin receptors have already shown an important role in resveratrol-uptake in SW480 cells [26], which we also used in the present work.

NF-kB has already been reported to be an active player in integrin expression, tumor survival, and malignant proliferation [44,45]. In addition, it has been repeatedly published that pro-inflammatory TME simultaneously induces phosphorylation of the transcription factor NF-kB and tumor-promoting NF-kB-governed proteins implicated in proliferation, metastasis, growth, and apoptosis of CRC cells [46,47]. Moreover, FAK has been shown to be a receptor tyrosine kinase that has an essential function in the intracellular signaling of integrins [18,19,40]. Therefore, to obtain more information about the underlying mechanism, we screened for NF-kB signaling in this work and found that p65-NF-kB, FAK (downstream of β1-integrin), and cyclin D1 (protein regulating cell cycle progression), strongly and at the same time, caspase-3 (apoptosis) was poorly expressed in TME. In numerous previous publications by our group, we compared the expression of these proteins in the TME of the cell lines also used in this work with a basal control without TME, confirming the notion that the described protein expressions are significantly induced in the TME [30,46,48,49,50]. In parallel, blocking the β1-integrin receptor by the antibody against β1-integrin but not against other integrin chains, the β5-integrin antibody, or by treatment with the integrin epitope suppressor RGD, but not with RGE peptides, led to inhibition of resveratrol-depressed p65-NF-kB, p-FAK, and cyclin D1 expression, and resveratrol-activated caspase-3. This finding suggests that this so-called central signaling cascade is engaged in the action of TME and β1-integrin-linked signaling is participating in the anti-tumorigenic effects of resveratrol in CRC cells. Furthermore, these outcomes are quite in line with past findings which have shown that FAK is an important regulating signaling molecule throughout the process of integrin-mediated signaling in various types of tumor cells, and plays a fundamental role in survival and invasion [51,52]. Indeed, integrins have been repeatedly reported to be a negative prognostic factor that also promotes cancer cell migration/invasion and metastasis [53,54,55], suggesting that they are possible cancer targets [25].

**Figure 6:**
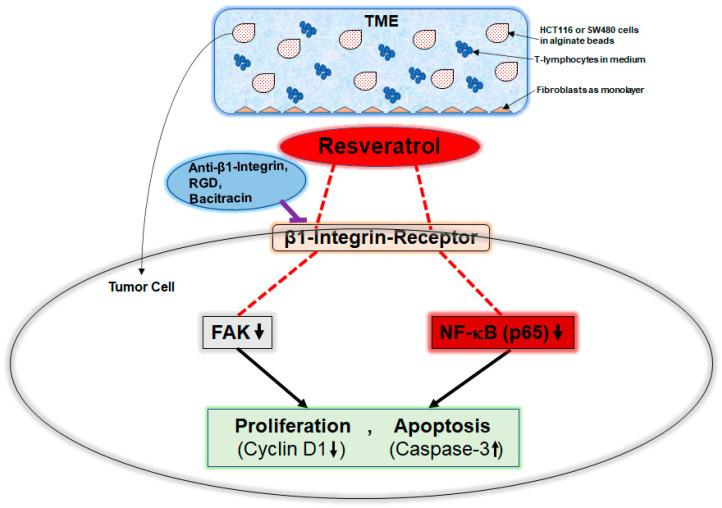
Working model demonstrating resveratrol-mediated anti-proliferative and anti-viability activity through modulation of the β1-integrin receptor in CRC cells in the pro-inflammatory TME.

Collectively, these observations illuminate that the resveratrol/β1-integrin/FAK/p65-NF-kB pathway may be an important underlying mechanism for resveratrol-promoted inhibition of CRC cell viability, growth, and proliferation in the TME. However, because our in vitro pro-inflammatory TME model is not sufficient to elucidate the precise physiological or pathological role of β1-integrin in vivo, further studies, possibly using transgenic mice with manipulated β1-integrin expression, are required (Figure 6). 

## 4. Materials and Methods

### 4.1. Antibodies and Chemicals

Anti-phospho p65-NF-kB (#MAB7226), anti-caspase-3 (#AF835), and anti-cyclin D1 (#MAB4314) were purchased from R&D Systems (Heidelberg, Germany). Anti-phospho-FAK (#558540) and anti-FAK (#610088) antibodies were from Becton Dickinson (Heidelberg, Germany). Anti-β-actin (#A4700) antibodies, MTT reagent (3-(4,5-dimethylthiazol-2-yl)-2,5-diphenyltetrazolium bromide), DAPI, resveratrol, bacitracin, and alginate were from Sigma-Aldrich (Taufkirchen, Germany). Monoclonal antibodies to β1-integrin (specifically recognizing the active conformation) are from BD Biosciences (#610468) (Monoclonal, 18/CD29) (San Diego, CA, USA). Neutralizing monoclonal antibodies against β1-integrin (#sc-374429), β5-integrin (#sc-374429), and normal mouse IgGs (#sc-2025) were from Santa Cruz (#sc-398214) (CA, USA). RGD (Arg-Gly-Asp) and RGE (Arg-Gly-Glu) peptides were obtained from Bachem (Torrance, CA, USA). Secondary rhodamine-coupled antibodies were from Dianova (Hamburg, Germany). Sheep anti-mouse and sheep anti-rabbit alkaline phosphatase-linked secondary antibodies were from Millipore (Schwalbach, Germany). RGD/RGE peptides were prepared by dilution in serum-free medium to minimize the effects of serum proteins. Resveratrol was prepared as a 100 mM stock in ethanol and further diluted in cell culture medium for experimental investigations. 

Cell culture medium from Seromed (Munich, Germany) consisting of Dulbecco’s modified Eagle’s medium/Ham’s F-12 (1:1) were completed with 3% FBS (fetal bovine serum, serum-starved) or 10% FBS, 1% glutamine, 1% penicillin/streptomycin solution (10,000 IU/10,000 IU), 75 μg/mL ascorbic acid, 1% essential amino acids, and 0.5% amphotericin B solution.

### 4.2. Cancer Cells, T-Lymphocyte, and Fibroblast Cell Growth Culture

HCT116 and SW480 are human colorectal cancer cell strains that differ in their KRAS mutation, and HCT116, among others, showed faster growth than SW480 [56]. Both CRC cell lines and MRC-5, a human fibroblast cell strain, were acquired from the European Collection of Cell Cultures (Salisbury, UK). T-cell leukemia cells Jurkat (ACC 282), THP-1 (ATCC^®^ TIB-202™), a human T-lymphocyte cell strain, were purchased from the Leibniz Institute (DSMZ-German Collection of Microorganisms and Cell Cultures). All cell strains were cultured under routine culture conditions at 37 °C and 5% CO_2_ in T175 flasks until a confluence of 70% was reached. HCT116, SW480, and MRC-5 cells grow as monolayers, whereas Jurkat cells freely float in suspension. Before starting an experiment, cells underwent three rinses with medium containing serum (3% FBS) and were pre-incubated in the same medium for 30 min. 

### 4.3. Tumor Microenvironment and Study Design

The purpose of this research was to evaluate the anti-cancer value of resveratrol via β1-integrin and to test different β1-integrin inhibitors in a pro-inflammatory TME in vitro, simulating a pro-inflammatory tumor situation in vivo. For this purpose, a model was set up with 3D-alginate beads as reported in our previous work [30,46]. 

As “basal control”, HCT116 or SW480 cells encapsulated in alginate matrix, were cultivated by itself in whole-cell culture medium. To accomplish the pro-inflammatory multicellular TME, fibroblasts were seeded as monolayers (20,000/well) and incubated in whole-cell culture medium containing 10% FBS. Subsequently, HCT116 or SW480 cells in 3D-alginate beads were added with 20,000 Jurkat cells/well (T-lymphocytes) to petri dishes containing the fibroblasts in serum-starved medium (3% FBS), as described before [30,46,50]. This composition of CRC-alginate beads, Jurkat cells, and fibroblasts served as “TME control”. TME cultures were either not treated, or treated with resveratrol (1, 5 µM) in the presence or absence of IgG (1, 2, 5 µg/mL), the functional blocking anti-β1-integrin antibody (1, 2, 5 µg/mL), anti-β5-integrin antibody (5 µg/mL), RGD peptide (1, 2, 5 µM), RGE peptide (5 µM), and bacitracin (1, 5 µM) for 10–14 days. 

### 4.4. Alginate Bead Culture

Human colon cancer cells (HCT116 or SW480) were embedded as beads in a sterile alginate suspension (2% in 0.15 M NaCl) as reported in our previous works [30,46,48,50]. For this purpose, CRC cells were counted, resuspended in alginate (1 Mio. CRC cells/1 mL alginate), and added dropwise to CaCl_2_ (100 mM) solution. After 10 min of polymerization, the beads were washed three times with NaCl (0.15 M) solution, then two times with cell culture medium (10% FBS), and before transfer to the investigation well-plates, beads were incubated with serum-starved medium (3%) for 30 min.

### 4.5. MTT Assay

To estimate the vitality and thus indirectly, the proliferation potential of HCT116 and SW480 cells in TME, the cells were detached from alginate, and an MTT assay was carried out as explained in detail previously [57,58]. To ensure that only the properties of the CRC cells were measured, each individual CRC alginate bead (size: ca. 0.5 cm) was first removed from the original experimental plates using bent tweezers, transferred to new plates containing fresh buffer (Hanks salt solution), and carefully washed on a shaker with gentle waving. This procedure was repeated at least three times (5 min each time) to ensure that no Jurkat cells (size: ca. 12 µM) adhered to the alginate beads. Each time, the CRC-alginate beads were observed under a phase-contrast microscope to check whether Jurkat cells adhered to the alginate beads. When it was 100% certain that no Jurkat cells were visible, the pure CRC-alginate beads were prepared for further processing. The clean CRC-alginate beads were then dissolved in sodium citrate (55 mM) for 30 min. After centrifugation and removal of sodium citrate and alginate residues, cells were washed with Hanks salt solution, resuspended in modified cell culture medium (3% FBS, without phenol red, without vitamin C) and distributed to a 96-well-plate with cell suspension (100 µL) and MTT solution (10 µL) to each well. The reaction was stopped by adding 100 µL of MTT solubilization solution (10% Triton x-100/acidic isopropanol) to each well after 3 h. The Optical Density (OD) was measured at 550 nm (OD550) using a 96-well-plate multi- scanner ELISA reader from Bio-Rad (Munich, Germany). At no time during the entire experiment, collection, and evaluation was there a risk of contamination with other cell types, as there was no direct contact.

### 4.6. Proliferation and Colony Formation 

As described in the study design section, HCT116 were cultured in alginate beads for 10–14 days. Treatment-dependent proliferation differences of CRC cells were observed, and their colonosphere formation in the alginate beads was quantified by counting 20 microscopic fields using a Zeiss Axiovert 40 CFL microscope (Oberkochen, Germany) as explained in detail previously [58]. Images from all different treatments were stored digitally and statistically evaluated.

### 4.7. Immunofluorescence Study

The TME was modified to perform immunofluorescence; 6-well-plates with fibroblast monolayers on the bottom and Jurkat cells in suspension were used. CRC cells were seeded on glass coverslips as a monolayer with 6000 cells/cover glass. After 24 h of incubation in 10% FBS cell culture medium, the glass plates were placed on small steel mesh bridges in the 6-well-plates as a basal control, or as TME with fibroblasts on the bottom, and 10,000 T-lymphocytes per mL of cell culture medium. Cells were incubated in basal control or TME for 24 h before treatment (5 µM resveratrol and/or 2 µg anti-β1-integrin) began. Immunofluorescence experiments were carried out as detailed in our earlier reports [30], where the dilution was 1:80 for the primary antibody and 1:100 for the secondary antibody. This experimental setup ensured exclusive labeling of HCT116 and SW480 CRC cells after removal of glass coverslips from the TME, as the steel bridges provided a distance to the fibroblast monolayer and the T-lymphocytes, which were floating in the cell culture medium and remained in the well-plates.

### 4.8. Western Blot Analysis

After 10–14 days of 3D-cultivation, the HCT116-alginate beads were separated from the TME, ensuring that the subsequent Western blot results were exclusively related to CRC cells. Therefore, the alginate beads, which contained only HCT116 cells, as described before, were removed from the experimental well-plates, transferred to a new well-plate, and carefully washed. Then, HCT116 were dissolved from alginate in sodium citrate solution and resuspended in lysis buffer (50 mM Tris-HCl, pH 7.2; 150 mM NaCl; 1% (*v*/*v*) Triton x-100; 1 mM sodium orthovanadate; 50 mM sodium pyrophosphate; 100 mM sodium fluoride; 4 µg/mL pepstatin A; 1 mM PMSF). After 30 min of centrifugation, the liquid supernatant was frozen at −80 °C. Standard Western blot assay was carried out as detailed in our earlier paper [46,48,58]. Briefly, proteins were separated by SDS-PAGE with a transblot apparatus from Bio-Rad (Munich, Germany). After pre-incubation in milk-powder-based blocking buffer (PBS, 5% milk-powder, 0.1% Tween 20), nitrocellulose membranes were incubated with primary antibodies (dilution 1:10,000) overnight and with secondary antibodies (dilution 1:10,000) for 90 min. The antibodies used are described in detail in “Antibodies and Chemicals” section and β-actin served as loading control. Bindings were quantified by densitometry using the program Quantity One from Bio-Rad (Munich, Germany).

### 4.9. Statistical Evaluation

Our studies were conducted in three separate assays with three different control samples. Results were analyzed by an unpaired Student’s t-test and by one-way ANOVA followed by a post hoc test to compare the parameters of each group. A *p* value of <0.05 indicates statistically significant differences.

## 5. Conclusions

Our results show for the first time that β1-integrins are overexpressed in TME-stimulated CRC cells, and resveratrol has a modifying impact on its expression and distribution on the tumor cell surface. It was shown here that resveratrol-promoted suppression of CRC cell proliferation, viability, and survival in TME occurs through down-regulation of FAK, p65-NF-kB, cyclin D1 activation, and up-regulation of caspase-3. As these effects can be reversed by anti-β1-integrin or RGD peptides, we conclude that resveratrol uses β1-integrin receptors as one of its major transmission molecules in its modulatory signal transduction in tumor cells (Figure 6). These results suggest a possible therapeutic implication of β1-integrins being a target for the development of anti-tumor drugs containing resveratrol or resveratrol-like natural components with the potential to inhibit viability as well as proliferation and induce apoptosis of CRC tumor cells. Further investigation of the role of β1-integrin in tumorigenesis in vivo will help to understand the potential therapeutic value of β1-integrin in the future treatment of colorectal cancer.

## Data Availability

All data are available in the manuscript.
